# Differences in Expression of Mitochondrial Complexes Due to Genetic Variants May Alter Sensitivity to Radiation-Induced Cardiac Dysfunction

**DOI:** 10.3389/fcvm.2020.00023

**Published:** 2020-03-05

**Authors:** Rachel A. Schlaak, Anne Frei, Gopika SenthilKumar, Shirng-Wern Tsaih, Clive Wells, Jyotsna Mishra, Michael J. Flister, Amadou K. S. Camara, Carmen Bergom

**Affiliations:** ^1^Department of Pharmacology & Toxicology, Medical College of Wisconsin, Milwaukee, WI, United States; ^2^Department of Radiation Oncology, Medical College of Wisconsin, Milwaukee, WI, United States; ^3^Medical Scientist Training Program, Medical College of Wisconsin, Milwaukee, WI, United States; ^4^Department of Medicine, Medical College of Wisconsin, Milwaukee, WI, United States; ^5^Department of Obstetrics and Gynecology, Medical College of Wisconsin, Milwaukee, WI, United States; ^6^Electron Microscope Facility, Department of Microbiology & Immunology, Medical College of Wisconsin, Milwaukee, WI, United States; ^7^Department of Anesthesiology, Medical College of Wisconsin, Milwaukee, WI, United States; ^8^Department of Physiology, Medical College of Wisconsin, Milwaukee, WI, United States; ^9^Cardiovascular Center, Medical College of Wisconsin, Milwaukee, WI, United States; ^10^Cancer Center, Medical College of Wisconsin, Milwaukee, WI, United States

**Keywords:** radiation, radiation-induced heart damage, mitochondria, consomic rats, oxidative phosphorylation, echocardiogram, cardiotoxicity

## Abstract

Radiation therapy is received by over half of all cancer patients. However, radiation doses may be constricted due to normal tissue side effects. In thoracic cancers, including breast and lung cancers, cardiac radiation is a major concern in treatment planning. There are currently no biomarkers of radiation-induced cardiotoxicity. Complex genetic modifiers can contribute to the risk of radiation-induced cardiotoxicities, yet these modifiers are largely unknown and poorly understood. We have previously reported the SS (Dahl salt-sensitive/Mcwi) rat strain is a highly sensitized model of radiation-induced cardiotoxicity compared to the more resistant Brown Norway (BN) rat strain. When rat chromosome 3 from the resistant BN rat strain is substituted into the SS background (SS.BN3 consomic), it significantly attenuates radiation-induced cardiotoxicity, demonstrating inherited genetic variants on rat chromosome 3 modify radiation sensitivity. Genes involved with mitochondrial function were differentially expressed in the hearts of SS and SS.BN3 rats 1 week after radiation. Here we further assessed differences in mitochondria-related genes between the sensitive SS and resistant SS.BN3 rats. We found mitochondrial-related gene expression differed in untreated hearts, while no differences in mitochondrial morphology were seen 1 week after localized heart radiation. At 12 weeks after localized cardiac radiation, differences in mitochondrial complex protein expression in the left ventricles were seen between the SS and SS.BN3 rats. These studies suggest that differences in mitochondrial gene expression caused by inherited genetic variants may contribute to differences in sensitivity to cardiac radiation.

## Introduction

Radiation therapy (RT) is used in over half of all cancer patients to treat malignancies and improve patient survival (1). RT can be administered to the thoracic region in treating chest tumors including Hodgkin lymphoma and breast and lung cancers. Despite advances in planning and delivering techniques (2–5), these techniques are not universally available and/or utilized by all providers (6), and RT to the thoracic region even with these techniques can still result in some exposure of the heart that can lead to cardiotoxicity (7, 8). Irradiation to the heart and surrounding vasculature may lead to toxicities including pericarditis, ischemic heart disease, myocardial fibrosis, cardiomyopathy, arrhythmias, and/or valvular abnormalities, collectively referred to as radiation-induced heart dysfunction (RIHD) (9–11). These normal tissue side effects may arise months to decades after RT, potentially leading to increased morbidity and mortality (12–14).

Cardiomyocytes are the most abundant cell type in the heart occupying roughly 70–85% total volume, and ~30% of the heart volume consists of cardiomyocyte mitochondria (15–17). The heart demands very high levels of adenosine triphosphate (ATP) for healthy function (18), and therefore mitochondrial function is crucial in maintaining heart health by coupling respiration with oxidative phosphorylation to generate ATP (7, 19, 20). Mitochondria are known to have roles in metabolism, cell death, and stress responses including combating reactive oxygen species (ROS). In addition to causing direct effects to DNA that may lead to cell death, radiation also causes indirect effects including the production of ROS. The mitochondria function to protect against ROS-induced cellular damage, and therefore, mitochondria play a role in protecting the normal heart tissue against radiation induced toxicity (21).

We previously reported that the inbred Dahl salt-sensitive/Mcwi (SS) rat strain was more sensitive to localized image-guided cardiac radiation than the Brown Norway (BN) strain, and that substitution of chromosome 3 from the BN strain into the SS background (SS.BN3 consomic rats) confers dramatic resistance to radiation-induced cardiac dysfunction when compared to the SS strain (22). Consomic chromosome substitution studies can be used to map complex genetic modifiers of pathophysiologic phenotypes (23–26). In our previous consomic rat study with the SS strain that was relatively sensitive to localized cardiac radiation when compared to the SS.BN3 consomic strain, the top genetic pathways differentially expressed between SS and SS.BN3 consomic rat ventricles 1 week after radiation included mitochondrial-related genes (22). However, expression of mitochondrial genes was not measured in unirradiated SS and SS.BN3 rat hearts, and protein expression of mitochondrial complexes was not examined. There is a need to better understand the mechanisms of mitochondrial dysfunction that may lead to RIHD. Here we examined changes in gene expression of all mitochondria-encoded genes and nuclear-encoded mitochondria oxidative phosphorylation complex genes between the sensitive SS and comparatively resistant SS.BN3 rat hearts that were not treated with radiation (sham treated). We also examined mitochondrial morphology using transmission electron microscopy, as well as the protein levels of mitochondrial oxidative phosphorylation complexes in isolated mitochondria from the left ventricles of SS and SS.BN3 rats after localized cardiac radiation. These results suggest that genetic changes can lead to altered expression of mitochondrial oxidative phosphorylation complexes that may contribute to differences in responses to localized cardiac irradiation. Better understanding of the role of mitochondrial dysfunction in RIHD may lead to targeted therapeutics to protect and/or mitigate RIHD while maintaining therapeutic effects of radiation therapy.

## Materials and Methods

### Rats and Irradiation Procedure

The rat cardiac irradiation procedure has been reported elsewhere (22). In brief, female SS and SS.BN3 rats [Medical College of Wisconsin (23)] aged 10–12 weeks were randomized into different treatment groups. Animals were anesthetized with 3% isoflurane and given localized heart irradiation using a the high-precision image-guided X-RAD SmART irradiator (Precision X-Ray, North Branford, CT). A 24 Gy × 1 fraction was given to the isocenter of the heart, with equally weighted anterior-posterior and 2 lateral beams (1:1:1, 225 kVp, 13 mA, 0.32 mm Cu, 2.69 Gy/min) using a 1.5 cm collimator. Pilot V1.8 Imaging Software (University Health Network, Toronto, Canada) was used to create two-dimensional projections over 360° to provide CT scans in sagittal, coronal, and axial views, with each projection on the heart centered to fit into the collimator. Monte Carlo-based treatment planning was utilized to calculate radiation dose (MAASTRO Radiotherapy Clinic, Netherlands). Age-matched sham-irradiated animals were included in the study. Animals were irradiated and housed in pathogen-free conditions with a 12:12 light:dark cycle and access to a standard diet (0.4% salt) and water. All procedures were performed according to the American Guidelines for the Ethical Care of Animals and approved by our Institutional Animal Care and Use Committee.

### Echocardiography

The echocardiogram procedure for rats has been reported elsewhere (22). In brief, echocardiography with M-mode was used to assess cardiac function on irradiated and sham treated rats at baseline, 3- and 5- months post-RT. An echocardiograph Vivid 7 with an 11-MHz M12L linear-array transducer and EchoPac software (General Electric, Wauwatosa, WI) was used to perform the examinations. Imaging was conducted in the short-axis view at mid-level of the left ventricle, by a sonographer with three consecutive heartbeats measured where the average was utilized for analyses (27, 28). For strain analysis, images were processed with EchoPac Q analysis software (General Electric, Wauwatosa, WI). A cardiac cycle was defined from peak one R wave to the peak of the following wave. The endocardial border was traced during an end-systolic frame in the short-axis view at mid-ventricle to calculate radial and circumferential strain. The computer produced a profile of radial (myocardial deformation toward the center) and circumferential (myocardial deformation along the curvature) strain percentage over time.

### RNA-Sequencing

The RNA-sequencing protocol was previously reported (22, 23). Briefly, total RNA was extracted by TRIzol (Thermo Fisher Scientific, Waltham, MA) from the left ventricle tissue of 11–13 weeks old female mock-treated SS and SS.BN3 rats (*N* = 4–5/group) from a group of rats matched to 1 week post-radiation rats (not reported here, but previously reported). For RNA-seq, a library preparation was made for each sample, indexed for multiplexing, and sequenced using an Illumina HiSeq2500 (Illumina, San Diego, CA). The Trim Galore program (v0.4.1) was used to trim bases with a Phred quality score <20 [https://www.bioinformatics.babraham.ac.uk/projects/trim_galore/]. The RSEM program function “rsem-prepare-reference” (v1.3.0) was used to extract the transcript sequences from the Rat genome (Rnor6.0, Ensembl release 98) (30) and to generate Bowtie2 indices (Bowtie2 v2.2.8) (31), followed by read alignment and expression quantification using the “rsem-calculate-expression” function. Differential expression (DE) analysis was performed using the Bioconductor package DESeq2 version 1.12.4 (29) to compute log2 fold changes and FDR-adjusted p-values. Statistical significance was determined at an FDR threshold of 0.05. Data were analyzed for molecular and functional pathway enrichment using the IPA tool (Qiagen). All raw sequencing data can be accessed from the Sequence Read Archive, BioProject ID PRJNA525087 (https://www.ncbi.nlm.nih.gov/bioproject/PRJNA525087).

### Transmission Electron Microscopy (TEM)

Rat left ventricle was harvested 1 week after 1 × 24 Gy cardiac RT or sham from adult female SS and SS.BN3 rats (*N* = 2–5/group) and fixed in 2.5% glutaraldehyde in 100 mM sodium cacodylate buffer pH 7.2. The samples were then post-fixed in 1% OsO_4_ on ice for 1 h, followed by dehydration in a graded methanol series, and an embedding in EPON 812 (EMS, Hatfield, PA). Ultra-thin sections (60 nm) were cut, stained with uranyl acetate and Reynolds lead citrate, and examined with a Hitachi H600 Transmission Electron Microscope (TEM) (Hitachi High Technologies America Inc., Pleasanton, CA). Representative images to assess cardiac mitochondrial morphology were captured at 20,000X magnification.

### Mitochondrial Isolation and Western Blot Analyses

Rat hearts were harvested 12 weeks after either 1 × 24 Gy localized cardiac radiation or sham treatment (22). Heart mitochondria isolation has previously been reported (32, 33). In brief, fresh heart tissue was minced in ice cold isolation buffer [200 mM mannitol, 50 mM sucrose, 5 mM KH_2_PO_4_, 5 mM 3-(N-morpholino) propanesulfonic acid, and 1 mM EGTA, with 0.1% bovine serum albumin, pH 7.15]. The minced tissue was homogenized in the presence of 5 U/ml protease (P5459, Sigma Life Science, St. Louis, MO) followed by differential centrifugation at 4°C. The final pellet was resuspended in isolation buffer and protein concentration was determined by the Bradford method. For Western blot analysis, isolated mitochondria were lysed using a RIPA buffer containing protease and phosphatase inhibitors, centrifugated, and the supernatant was collected. Total protein was assessed using a BCA Protein Assay Kit (23225, Thermo Scientific, Rockford, IL). Mitochondrial protein lysates were loaded and separated using SDS-PAGE and then transferred onto a PVDF membrane. The following antibodies were used in the present study using mitochondrial lysates: total OXPHOS rodent WB antibody cocktail (1:2500; ab110413; Abcam) and anti-COX IV antibody Mitochondrial Loading Control (1:5000; ab16056; Abcam).

### Statistical Analysis

Analyses of the western blotting were evaluated by a Student's *t*-test. Blots were imaged on ImageQuant LAS 4000 (GE Healthcare Life Sciences, Marlborough, MA), and analyzed using ImageQuant TL software (version 8.1.0.0). All western blotting results reported are representative of 3 technical replicates. The criterion for significance was *P* < 0.05. Data are reported as means ± SE. For our RNA-sequencing studies (22), power analysis was determined using a combination of simulated and experimental data approach previously described (34). We performed 100 simulations based on a RNAseq count data from our previous study (35). This analysis suggested that 4 replicates per group in a 2-group comparison would provide more than 90% power to detect genes differentially expressed at FDR 0.05 level. All power calculations and animal numbers for our studies were also performed by a non-biased statistician (S.-W.T.).

## Results and Discussion

**Figure 1:**
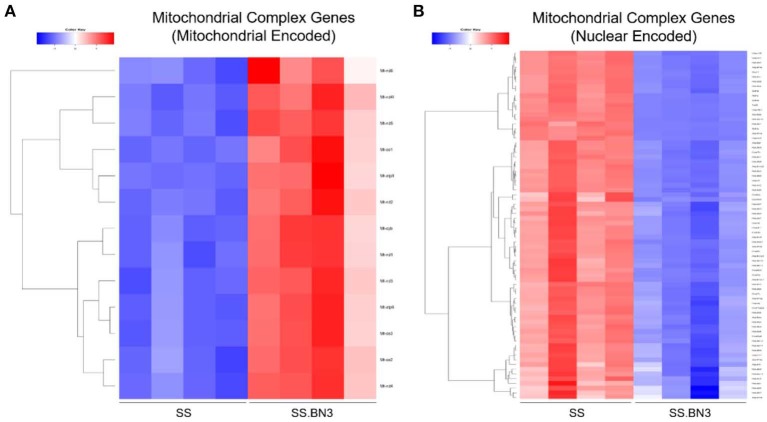
RNA-seq analysis of control SS and SS.BN3 hearts. Total RNA was extracted and RNA-seq was performed on RNA from the left ventricle tissue of adult 10–12 week old female SS and SS.BN3 rats harvested 1 week after mock treatment (*N* = 4/group). Differential expression analysis was performed, followed by generation of heat maps of **(A)** 13 mitochondrial encoded genes and **(B)** 74 nuclear encoded genes differentially expressed at FDR < 0.05 and involved in the mitochondrial complexes that drive oxidative phosphorylation.

**Figure 2:**
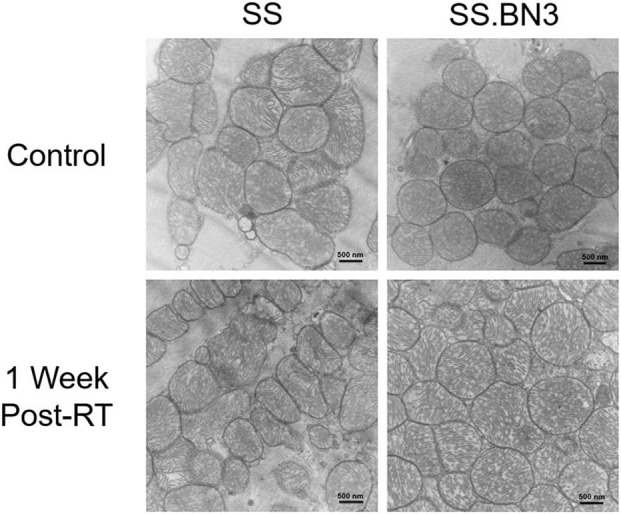
Representative TEM images revealed SS and SS.BN3 have no observed changes in mitochondrial morphology at 1 week post-radiation therapy (RT). Transmission electron microscopy (TEM) was performed on SS and SS.BN3 rat left ventricle tissue harvested 1 week after either 24 Gy RT or mock treatment (*N* = 2–5/group). No gross changes in mitochondria were seen between groups. Representative images from each condition are shown. Scale bar = 500 nm.

Previously, we have demonstrated that the SS rat strain is more sensitive to localized image-guided cardiac radiation that the SS.BN3 consomic rat strain, which differs only in substitution of chromosome 3 from the BN strain, as measured by pleural effusions, echocardiogram indices of left-sided heart failure and strain, as well as mortality. We also demonstrated that the SS and SS.BN3 strains had differentially expressed mitochondria-related genes in the left ventricle 1 week after radiation, as measured with RNA-sequencing (22). In this study, we examined the differential expression of oxidative phosphorylation genes from both the mitochondrial and nuclear genomes between SS and SS.BN3 left ventricles in rats 1 week after sham radiation treatment, at 11–13 weeks of age (*N* = 4/condition), which is the same time period reported previously after radiation (22). Of the mitochondrial-encoded genes coding for oxidative phosphorylation complexes, 13 of 13 genes are differentially expressed between SS and SS.BN3 rats at FDR < 0.05 (Figure 1A, Supplemental Table 1B). Expression of these genes was significantly higher in the protected SS.BN3 rats in comparison to the more sensitive SS rats. In addition, of the 80 nuclear encoded rat genes involved in encoding mitochondrial complexes I–V, 74 of 80 genes were differentially expressed between SS and SS.BN3 rats at FDR < 0.05 (Figure 1B, Supplemental Table 1A). Interestingly, these genes had higher expression in SS rats compared to the SS.BN3 rats. We subsequently examined whether there were changes in mitochondrial morphology between the SS and SS.BN3 rat left ventricles. TEM was performed on mitochondria isolated from rat hearts at 1 week post-radiation or sham treatment. Longitudinal views of tissue were examined for each condition, where total mitochondria and irregular shaped mitochondria were counted. This revealed no morphological differences between SS and SS.BN3, with representative images shown in Figure 2.

**Figure 3:**
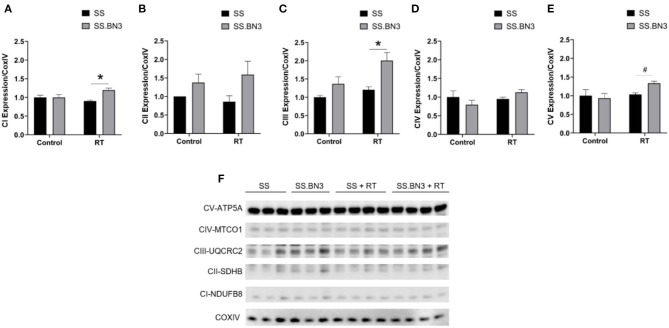
Oxidative phosphorylation complex expression in rat cardiac mitochondria. Subunits of the mitochondrial complex expression was visualized via Western blotting and quantified. These included **(A)** Nuclear-coded NDUFB8 Complex I-subunit, **(B)** Nuclear-coded SDHB Complex II-subunit, **(C)** Nuclear-coded UQCR2 Complex III-subunit, **(D)** Mitochondrial-coded MTCO1 Complex IV-subunit, and **(E)** Nuclear-coded ATP5A Complex V-subunit. All were measured and quantified from a Western blot from mitochondria lysates of rat hearts 12 weeks post-RT or sham treatment **(F)**. Representative blots are originated from different exposure times of the same blot using an antibody cocktail, and technical replicates of the Western blot were run 3 times total, with a representative blot and quantifications from one experiment shown. Values are expressed as means ± SEM normalized to their respective COX IV loading control, and then expressed as fold change relative to the SS sham treated; *n* = 3–4/ group; ^*^P < 0.05, ^#^*P* < 0.01. A Student's *t*-test was used to determine significance in SS vs. SS.BN3 control (lanes 1–3 and 4–6, respectively) and SS vs. SS.BN3 with RT (lanes 7–10 and 11–14, respectively).

**Figure 4:**
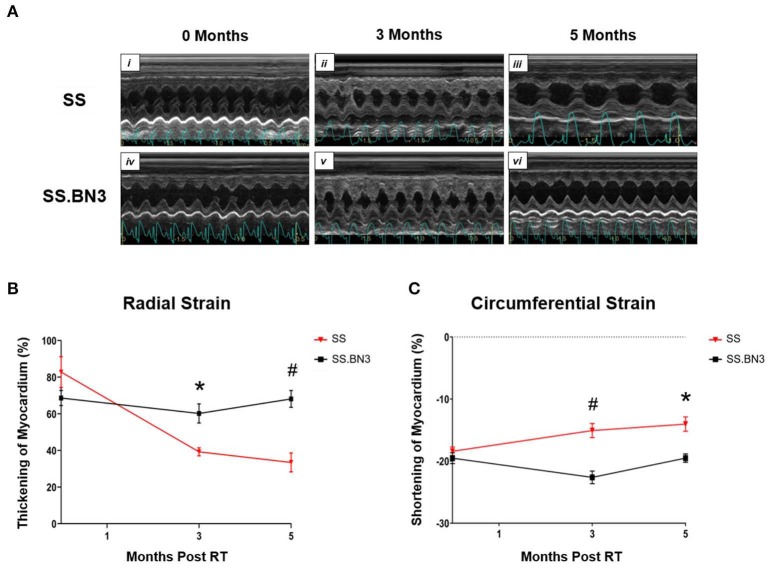
Echocardiograms indicated SS rats have decreased heart function compared with SS.BN3 rats after 24 Gy localized heart RT. **(A)** M-mode echocardiogram images of SS and SS.BN3 rats that received 24 Gy RT at baseline, 3 months, and 5 months post -RT. **(B)** Radial strain was lower in the SS rats at 3 and 5 months post-RT shown via decreased thickening of myocardium. **(C)** Circumferential strain also showed decreased function in SS vs. SS.BN3 at 3 and 5 months post-RT via decreased ability to contract, indicated by a smaller negative percentage. Values are means ± SEM. ^*^P < 0.01, ^#^*P* < 0.001.

Our data in Figure 1, along with previously published data (22), demonstrate that changes in gene expression of oxidative phosphorylation complex genes are differentially expressed in the left ventricles of both the non-irradiated rats and rats irradiated with a single dose of 24 Gy to the whole heart. However, the functional consequences of these changes at later time points had not been examined. We isolated mitochondria from SS and SS.BN3 rats (N of 3–4 per group) 12 weeks post-treatment with 24 Gy of localized heart radiation or sham (no radiation). We then performed Western blotting on the isolated mitochondria to examine protein expression of mitochondrial complexes I–V. This revealed no significantly significant changes between complex I–V in the unirradiated SS vs. SS.BN3 heart, but significant increases were seen in complexes I, III, and V in the SS.BN3 vs. SS hearts (Figures 3A–F, Supplemental Figure 1). Figure 3 shows representative results from 3 technical replicates of each Western blot. Protein expression levels were assess using NADH: Ubiquinone Oxidoreductase Subunit B8 (NDUFB8; complex I) (Figure 3A), Succinate dehydrogenase ubiquinone iron-sulfur subunit (SDHB; complex II) (Figure 3B), Ubiquinol Cytochrome C Reductase Core Protein 2 (UQCRC2; complex III) (Figure 3C), Mitochondrial Cytochrome C Oxidase I (MT-CO1; complex IV) (Figure 3D), and Mitochondrial ATP Synthase 5A (ATP5A; complex V) (Figure 3E). Representative Western blots of the mitochondrial lysates are shown in Figure 3F, *N* = 3–4/group. complexes I, III, and V showed increased expressions in the SS.BN3 vs. SS with RT lysates (complex I: *P* = 0.004, 2/3 blots significant; complex III: *P* = 0.02, all 3 blots were significant; CV: *P* = 0.004, all 3 blots were significant). There was also a trend in complex IV with increased expression in SS.BN3 vs. SS with RT (1/3 blots significant). Although the OXPHOS antibody cocktail consists of a mixture of five antibodies to detect the five different complex subunits, different subunits were quantified at different exposure times to be in the linear detection range, as shown in Figure 3F. The full blots at different exposure times are shown in Supplemental Figure 1. These results indicate that genetic changes in rat chromosome 3 can lead to significant changes in mitochondrial complex expression several weeks after high-dose cardiac radiation exposure, at a time when echocardiogram changes are seen demonstrating differences in left ventricular heart function between the SS and SS.BN3 rats (22). M-mode echocardiogram imaging, performed previously (22) demonstrated cardiac dysfunction in SS rats compared to SS.BN3 rats displaying hyperdynamic systolic function (Figure 4A). Analysis of both radial and circumferential strain at 3 and 5 months post-RT revealed the SS rat hearts had significantly decreased myocardium deformation, consistent with decreased systolic dysfunction (Figures 4B,C).

A number of studies have implicated mitochondrial changes in the development of cardiac dysfunction following radiation, both in pre-clinical models and in human studies (7, 36–39). In C57BL/6N mice that received sham, 0.2 Gy, or 2 Gy of heart radiation, functional and proteomic alterations were seen 4 weeks following irradiation. This included changes in proteins related to oxidative phosphorylation (36, 40–42). Functionally, partial deactivation of complexes I and III were observed in mice receiving 2 Gy of cardiac radiation. In a separate publication, this group also examined the long-term effects of cardiac radiation, finding that respiratory capacity was still reduced 40 weeks after 2 Gy of cardiac radiation (38). In separate studies, C57BL/6 mice treated with 8 or 16 Gy of cardiac radiation demonstrated increased free fatty acids and reduced levels of complexes I, III, and V (39). Studies of mitochondrial-related proteins in the left ventricles of decreased nuclear workers exposed to varying levels of radiation (external exposure ranges from 100 mcGy to >5 Gy) revealed dose-dependent reductions in complexes I, III, and V, and changes in complexes II and IV in those with the highest radiation exposures (42).

There are limitations from this study that should be acknowledged. We examined the effects of RIHD with the treatment dose of 1 × 24 Gy cardiac RT. We have previously reported similar cardiac trends by using a fractionated regimen of 9 Gy × 5 (22). The dosing regimen was determined based on previous studies of studying RIHD from cardiac RT in rats (7, 43–47). To better mimic cancer patient thoracic RT, future studies are needed with both partial heart irradiation and increased fractions of smaller daily radiation dose to more closely resemble the radiation exposure observed. In addition to cancer patients receiving thoracic RT, recent studies report the using 25 Gy cardiac RT in a single fraction to treat ventricular tachycardia (48, 49). Our rat model of cardiac RT is very relevant to this clinical model of treatment and could be further used to study side effects and biologic changes that occur from this high dose cardiac RT. Additional considerations include how these findings can be translated into future applications. Other than the 13 mitochondrial encoded genes, many genes involved in mitochondrial dysfunction, sirtuin signaling and cardiac hypertrophy were also found to be differentially expressed between SS and SS.BN3 rats (22). Candidates involved in these pathways as well as mitochondrial gene transcription, translation, and regulation could be further tested to investigate their roles in radiation-induced cardiotoxicity. The use of pharmacologic modulators of these pathways and transgenic models could also be pursued to further elucidate mechanisms of RIHD to prevent and/or mitigate effects observed in patients receiving radiation therapy.

In this current study, we demonstrate changes in the levels of oxidative phosphorylation complexes between genetically similar rats, differing only in the single nucleotide polymorphisms on chromosome 3, that demonstrate dramatic differences in the development of radiation-induced cardiotoxicity after localized radiation exposure to the heart (22). These results demonstrate that there are differences in gene expression of both mitochondrial-encoded and nuclear-encoded genes for the oxidative phosphorylation complexes in the left ventricles of unirradiated SS and SS.BN3 rats (Figure 1), as well as the left ventricles of SS and SS.BN3 rats 1 week after 24 Gy of localized cardiac irradiation (22). It is unclear why there are differences in the direction of differential expression of oxidative phosphorylation complex genes encoded by mitochondrial vs. nuclear genomes. In general, the mitochondrial genome is more likely to experience DNA damage than nuclear DNA following radiation due to the lack of protective effect from histones (50), as well as less efficient DNA repair (51, 52). However, as our results here demonstrate, differences in mitochondrial-encoded genes are seen between SS and SS.BN3 left ventricles even without radiation treatment (Figure 1). Although large numbers of mitochondrial genes are differentially expressed in SS vs. SS.BN3 rats in unirradiated and irradiated left ventricles, no gross changes in mitochondrial morphology were seen in the left ventricles 1 week after radiation or sham treatments (Figure 2). However, at a later timepoint of 12 weeks following 24 Gy of localized cardiac radiation, differences in expression of complex I, III, and V proteins were seen in isolated mitochondrial in the SS vs. SS.BN3 samples. Taken together, these results indicate that inherited genetic variants can lead to differences in oxidative phosphorylation gene expression that may contribute to differences in radiation-induced cardiac dysfunction.

## Data Availability Statement

The datasets generated for this study can be found in https://www.ncbi.nlm.nih.gov/sra, NCBI Accession No. PRJNA525087.

## Ethics Statement

The animal study was reviewed and approved by Medical College of Wisconsin Institutional Animal Care and Use Committee.

## Author Contributions

RS, MF, AC, JM, and CB conceived and designed the research. RS, AF, GS, and CW performed the experiments. RS, AF, CW, S-WT, and CB analyzed data and interpreted results. RS, AF, and S-WT prepared figures. RS and CB drafted the manuscript. RS, GS, JM, AC, and CB edited and revised manuscript. All authors approved the final version of manuscript.

### Conflict of Interest

MF currently is a Principal Research Scientist at Abbvie, but at the time of his contributions to the manuscript, he was employed at the Medical College of Wisconsin. CB receives research funding from Innovation Pathways, Palo Alto, CA. The remaining authors declare that the research was conducted in the absence of any commercial or financial relationships that could be construed as a potential conflict of interest.

## Supplementary Material

Supplemental Figure 1 Full immunoblots against OXPHOS antibody cocktail in heart mitochondrial lysates from SS and SS.BN3 rats with either 24 Gy RT or sham treatment at different exposure times of **(A)** 4 s for C-V, **(B)** 8 s for C-III, **(C)** 30 s for C-IV, and **(D)** 5 min for C-I and C-II.

Supplemental Table 1A Expression of Nuclear-Encoded Mitochondrial Complex Genes in SS.BN3 vs. SS Left Ventricles 1 Week After 24 Gy of Localized Heart Radiation.

Supplemental Table 1B Expression of Mitochondrial-Encoded Genes in SS.BN3 vs. SS Left Ventricles 1 Week After 24 Gy of Localized Heart Radiation and Differentially Expressed Mitochondrial Genes in SS versus SS-BN3 Left Ventricles Mitochondrial-Encoded Genes.
