# Supplementary figures and images for: Differences in Expression of Mitochondrial Complexes Due to Genetic Variants May Alter Sensitivity to Radiation-Induced Cardiac Dysfunction
Source: Front Cardiovasc Med. 2020 Mar 5;7:23. doi: 10.3389/fcvm.2020.00023 (PMC7066205; doi:10.3389/fcvm.2020.00023)

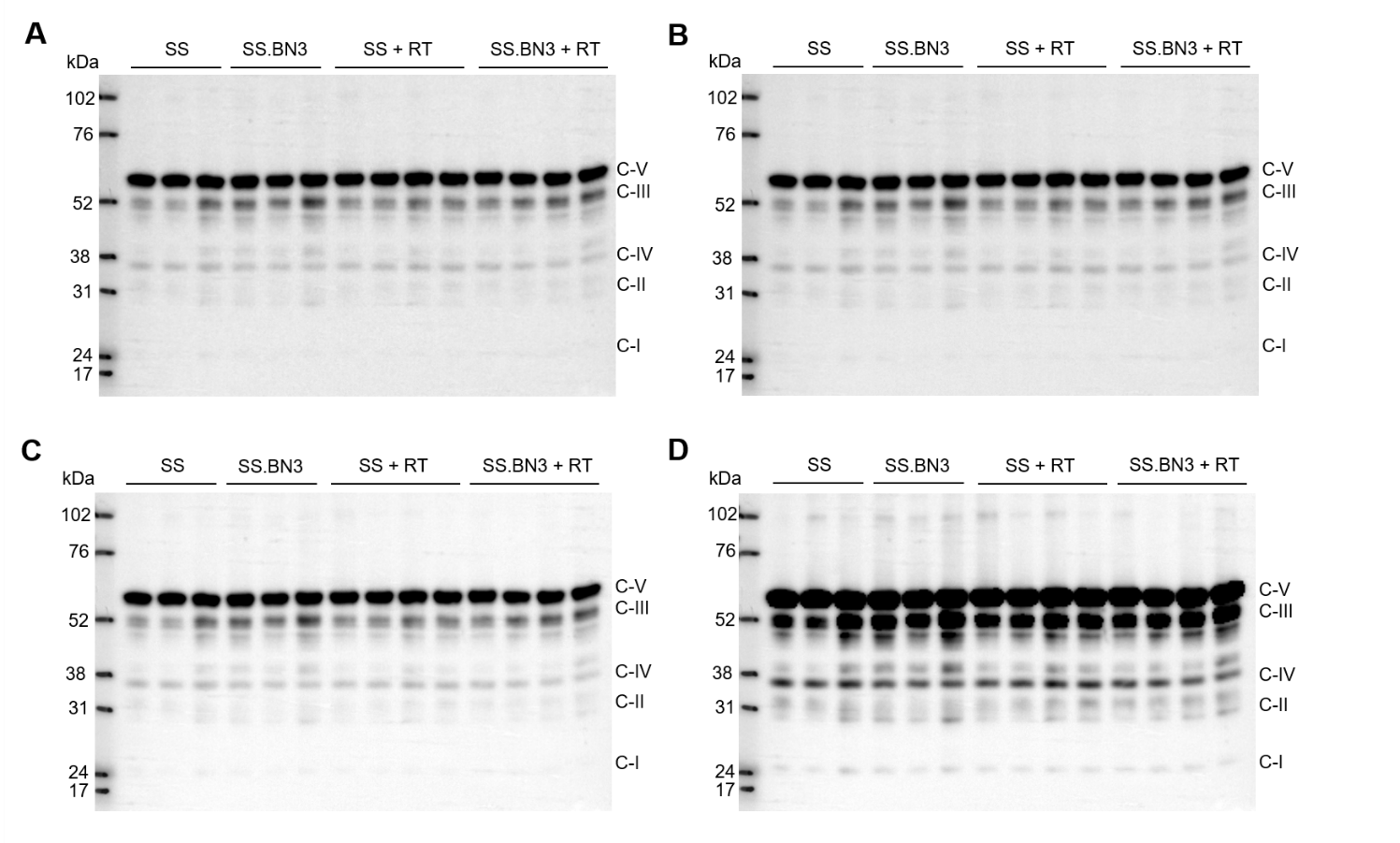

Supplement: Supplemental Figure 1 — Full immunoblots against OXPHOS antibody cocktail in heart mitochondrial lysates from SS and SS.BN3 rats with either 24 Gy RT or sham treatment at different exposure times of (A) 4 s for C-V, (B) 8 s for C-III, (C) 30 s for C-IV, and (D) 5 min for C-I and C-II. [file Image_1.TIF]
