# Supplementary material for: Differences in Expression of Mitochondrial Complexes Due to Genetic Variants May Alter Sensitivity to Radiation-Induced Cardiac Dysfunction
Source: Front Cardiovasc Med. 2020 Mar 5;7:23. doi: 10.3389/fcvm.2020.00023 (PMC7066205; doi:10.3389/fcvm.2020.00023)
Supplement: Supplemental Table 1A — Expression of Nuclear-Encoded Mitochondrial Complex Genes in SS.BN3 vs. SS Left Ventricles 1 Week After 24 Gy of Localized Heart Radiation. [file Data_Sheet_1.pdf]

Supplemental Table 1A. Expression of Nuclear-Encoded Mitochondrial Complex Genes in SS.BN3 versus SS Left Ventricles 1 Week After 24 Gy of Localized Heart Radiation.

Differentially Expressed Genes in SS versus SS.BN3 Left Ventricles<br>Nuclear-encoded Mitochondrial Complex Genes				
Symbol	Entrez Gene ID	Chromosome	Expr Log Ratio	Expr False Discovery Rate (q-value)
Atp5me		14	-0.855386253	0.026097496
Cox6a1	25282	12	-0.942094326	2.54E-14
Ndufv3	64539	20	-1.167884751	3.06E-07
Atp5pf	94271	11	-1.946448273	3.38E-27
Atp5mc3	114630	3	-2.561395584	3.28E-26
Atp5po	192241	11	-1.673777528	1.57E-12
Ndufb4	288088	11	-1.75533924	4.72E-16
Atp5f1b	171374	7	-2.871582953	1.98E-33
Sdhc	289217	13	-2.453035729	2.84E-124
Ndufa8	296658	3	-2.033450948	4.22E-52
Ndufa5	25488	4	-0.856508205	0.001200898
Ndufa7	299643	7	-1.83052046	6.92E-19
Atp5mc1	29754	10	-2.764657728	1.97E-14
Ndufa12	299739	7	-1.649584684	1.25E-12
Cox4i2	84683	3	0.1508469	0.511309637
Sdhb	298596	5	-2.221737952	1.34E-24
Ndufb11	299310	X	-2.016583236	1.20E-17
Ndufa6	315167	7	-1.729950495	2.35E-17
Ndufs3	295923	3	-2.44678498	3.65E-40
Ndufb9	299954	7	-1.541616741	3.86E-05
Cox6c	54322	7	-2.344787413	8.42E-27
Ndufs4	499529	2	-2.223161282	1.88E-35
Ndufb3	301427	9	-1.589034636	1.91E-10
Ndufs1	301458	9	-4.127417503	3.24E-275
Ndufb5	294964	2	-2.32220647	3.02E-22
Ndufc2	293130	1	-1.860643512	8.96E-17
Cyc1	300047	7	-2.620803582	7.37E-57
Uqcrh	366448	5	-2.360206435	5.10E-42
Atpif1	25392	5	-0.882812331	3.36E-08
Sdha	157074	1	-3.170298063	2.44E-251
Ndufb8	293991	1	-2.427293661	1.46E-46
Ndufb10	681418	10	-2.007510035	9.48E-27
Atp5f1d	245965	7	-2.166173592	3.02E-36
Atp5mc2	171082	7	-1.747232108	1.83E-38
LOC100911417	100911417	2	-3.537089528	7.90E-241

Ndufa10	316632	9	-3.034136769	3.39E-109
Cox5b	94194	9	-2.12911377	1.91E-09
Uqcr11	690848	7	-1.519113392	7.50E-10
Atp5f1a	65262	18	-3.92926468	7.71E-122
Ndufs8	293652	1	-1.961432302	2.89E-20
Ndufa2	291660	18	-1.708199268	7.68E-12
Cox4i1	29445	19	-1.878329295	8.33E-17
Ndufv1	293655	1	-2.493156607	9.30E-43
Ndufab1	293453	1	-2.953223559	1.19E-47
Uqcrfs1	291103	17	-3.491615347	7.23E-113
Cox5a	252934	8	-2.90108086	2.63E-34
Taf3	116550	17	-3.275972006	6.33E-177
Cox6a2	25278	1	-2.076041801	3.20E-05
LOC100911483	100911483	16	-1.349327753	0.000265323
Cox8a	171335	1	-1.024358217	3.88E-14
Sdhd	363061	8	-2.767605428	1.42E-89
Cox6b1	688869	1	-2.058434822	9.22E-31
Ndufb6	297990	5	-2.478357183	3.14E-42
Ndufs7	362837	7	-1.430954879	6.00E-14
Uqcrb	362897	7	-1.725370083	5.58E-17
Ndufb2	362344	4	-0.981681574	3.06E-05
Ndufs5	362588	5	-1.456391305	3.44E-14
Atp5mf	690441	12	-1.773269603	3.27E-23
Ndufb7	361385	19	-0.951524446	0.012059338
Atp5l	300677	8	-1.72717547	1.53E-06
Cox7c	100188937	2	-2.009066265	1.68E-16
Uqcrc1	301011	8	-2.955716001	5.89E-51
Uqcrc2	293448	1	-3.294789394	2.58E-264
Ndufc1	689938	2	-2.359630348	8.72E-24
Ndufs2	289218	13	-2.792941564	2.60E-43
Ndufa1	108348144	X	-1.356186305	0.007201069
Ndufv2	81728	9	-2.444826711	1.09E-29
Cox7a2l2	29507	8	-1.70754915	1.12E-11
Cox7a1	100910772	1	0.300908829	0.343580708
Uqcrq	497902	10	-1.522786644	1.97E-05
Ndufa11	301123	9	-1.525974579	6.49E-10
Atp5f1e	245958	3	-1.548052137	6.69E-12
Cox7b	303393	X	-2.395873574	1.33E-21
Uqcr10	685322	14	-2.66833371	3.42E-51
Ndufa3	691001	1	-1.751903054	4.61E-13
Ndufa9	362440	4	-2.607866894	3.95E-31

Supplemental Table 1B. Expression of Mitochondrial-Encoded Genes in SS.BN3 versus SS Left Ventricles 1 Week After 24 Gy of Localized Heart Radiation.

Differentially Expressed Mitochondrial Genes in SS versus SS-BN3 Left Ventricles Mitochondrial-encoded Genes				
**Symbol**	**Entrez Gene ID**	**Chromosome**	**Expr Log Ratio**	**Expr False Discovery Rate (q-value)**
Mt-nd6	26203	MT	1.110277231	7.17E-12
Mt-nd4	26201	MT	1.158822309	7.04E-19
Mt-nd5	26202	MT	1.313418587	1.05E-22
Mt-co2	26198	MT	1.099804942	1.55E-15
Mt-nd1	26193	MT	1.075422829	1.26E-15
Mt-cox3	26204	MT	0.94898068	1.62E-14
Mt-nd2	26194	MT	1.16362266	3.28E-21
Mt-nd4l	26200	MT	1.359098464	4.44E-29
Mt-cyb	26192	MT	1.00441361	7.01E-17
Mt-atp6	26197	MT	1.008376595	1.41E-15
Mt-atp8	26196	MT	1.141528399	1.41E-19
Mt-nd3	26199	MT	0.956194609	2.99E-15
Mt-co1	26195	MT	1.018705868	6.86E-18
